# Supplementary figures and images for: Arp2/3 complex activity enables nuclear YAP for naïve pluripotency of human embryonic stem cells
Source: eLife. 2024 Sep 25;13:e89725. doi: 10.7554/eLife.89725 (PMC11509671; doi:10.7554/eLife.89725)

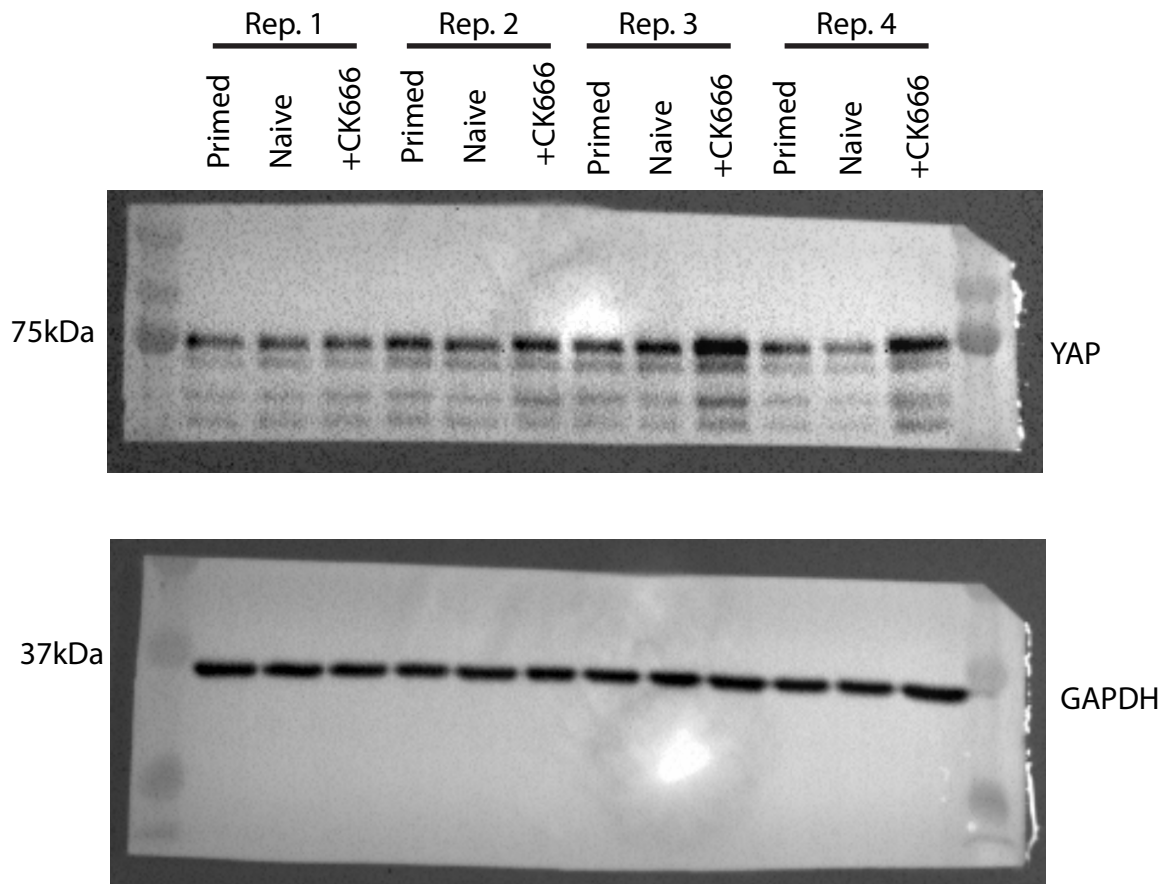

Supplement: Figure 3—figure supplement 1—source data 1. [file elife-89725-fig3-figsupp1-data1.zip › Figure 3 - figure supplement 1 - source data 1.pdf]

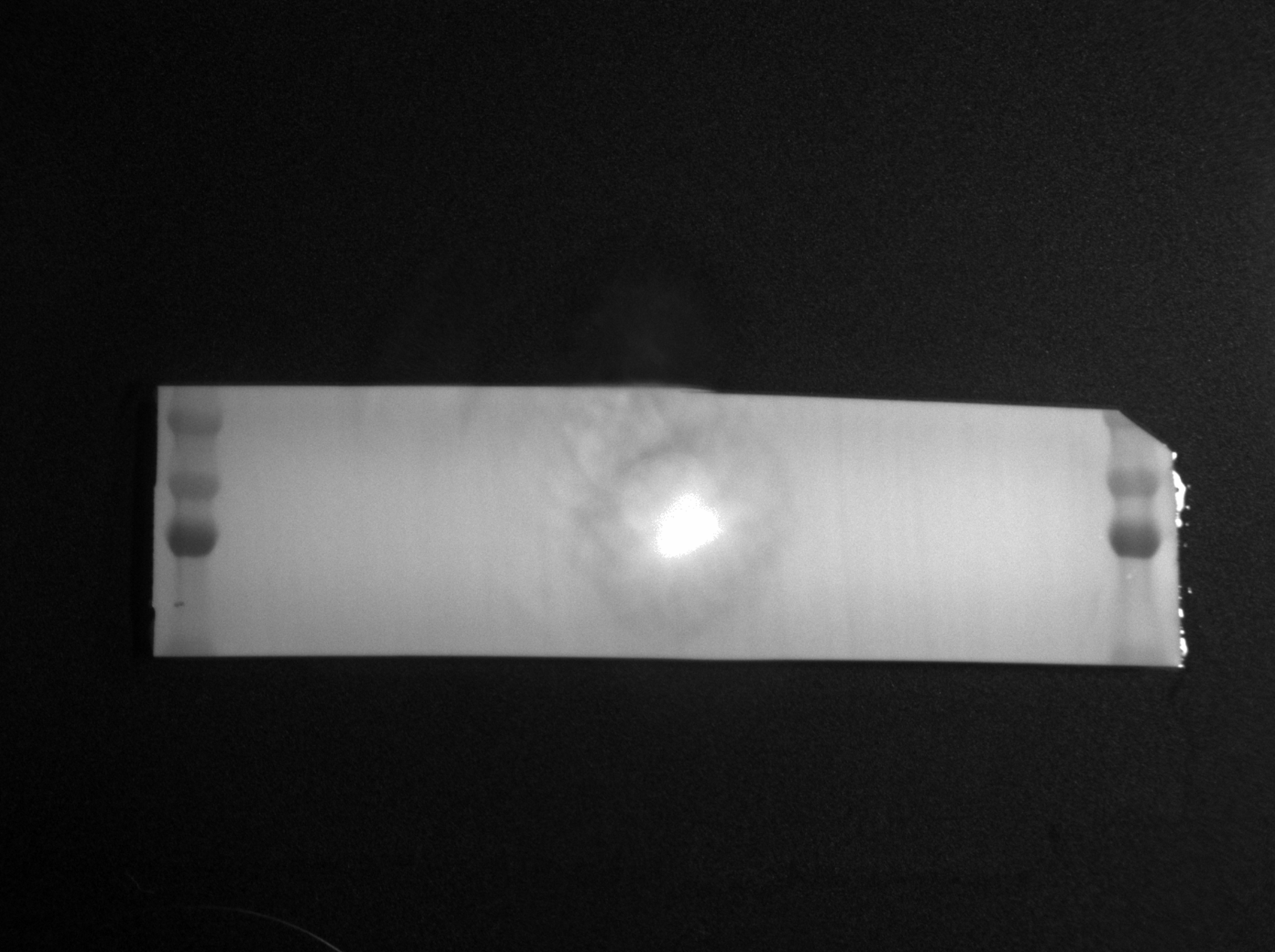

Supplement: Figure 3—figure supplement 1—source data 2. [file elife-89725-fig3-figsupp1-data2.zip › YAP ladder.tif]

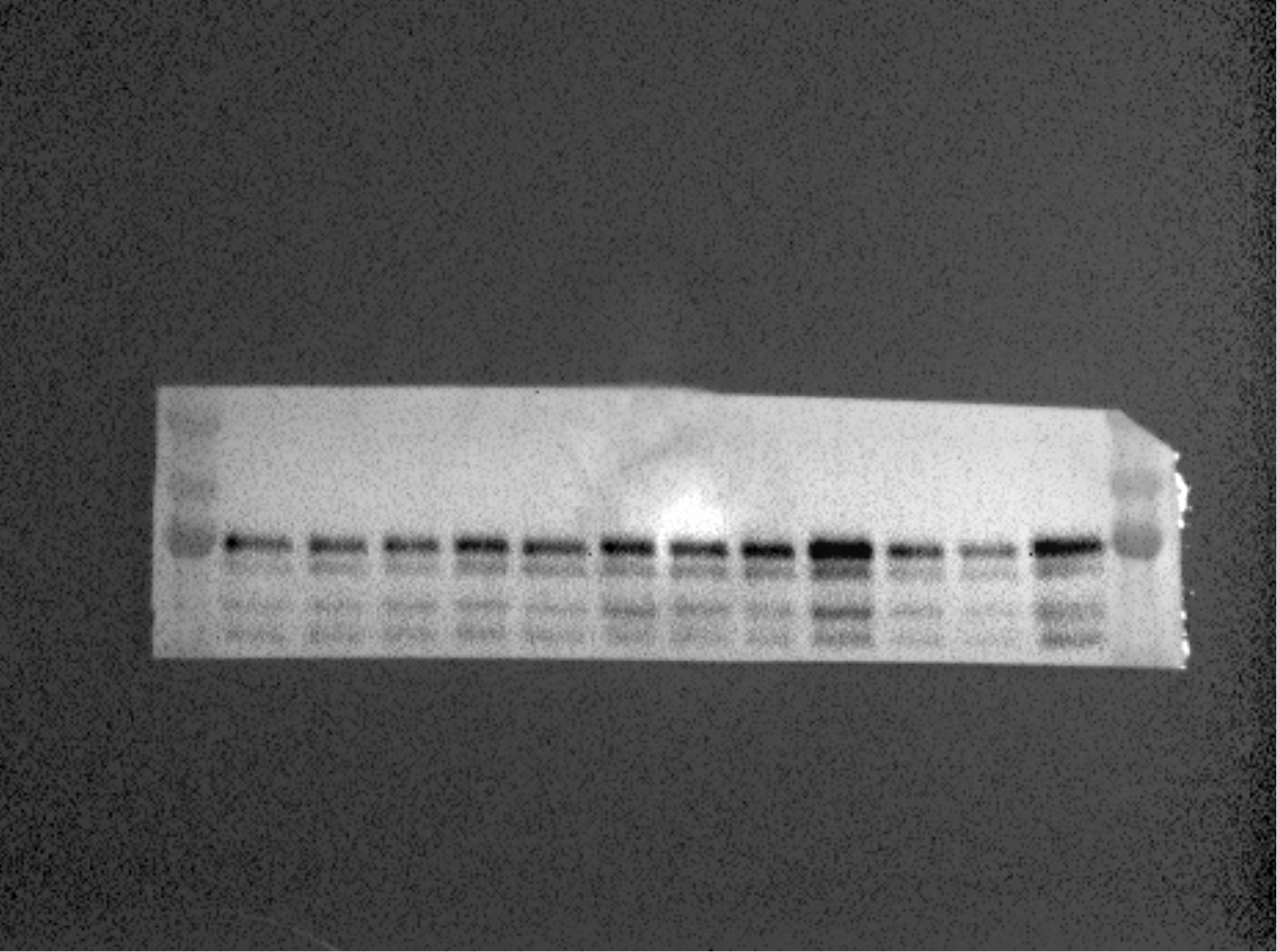

Supplement: Figure 3—figure supplement 1—source data 2. [file elife-89725-fig3-figsupp1-data2.zip › YAP merge.tif]

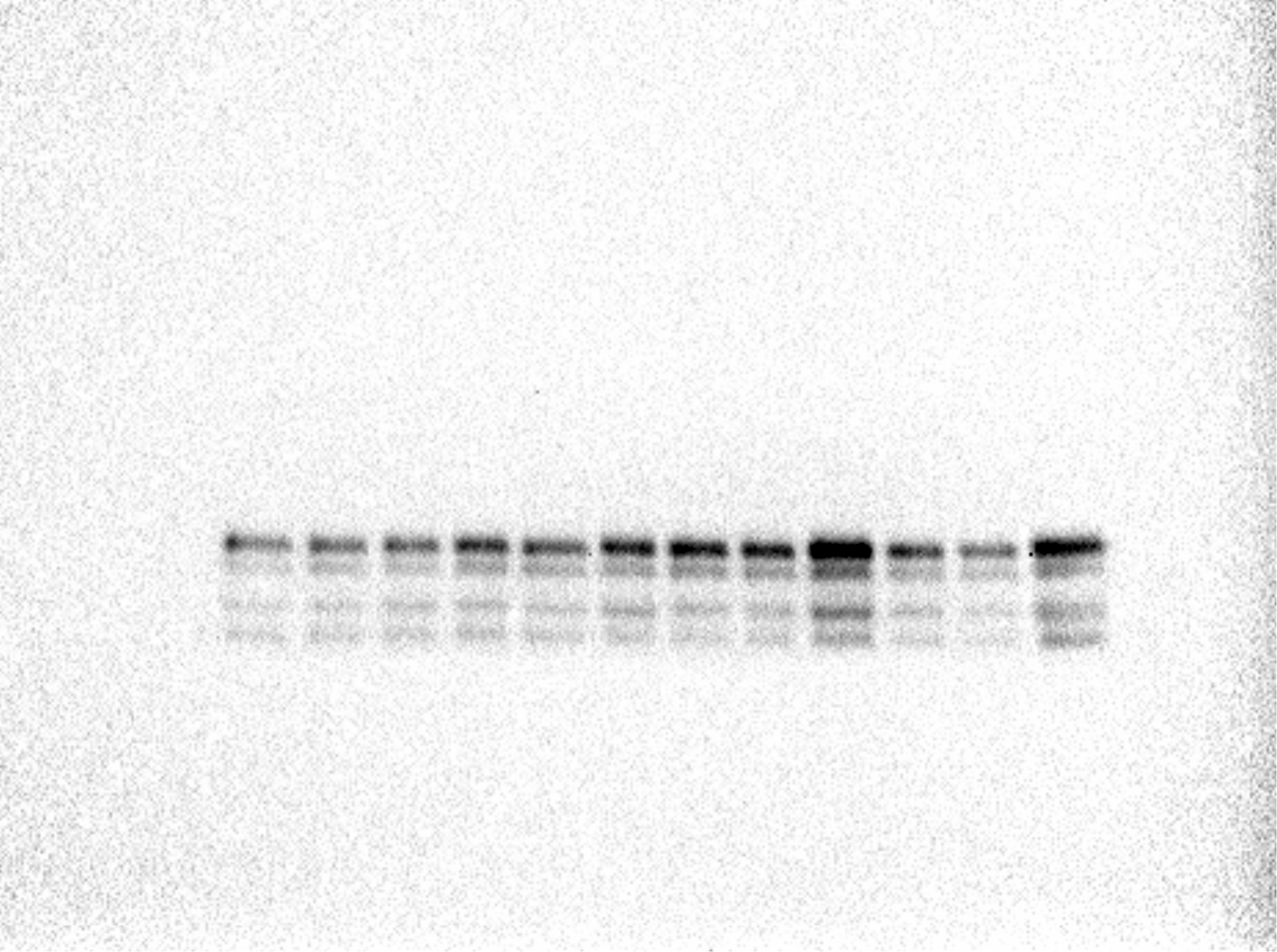

Supplement: Figure 3—figure supplement 1—source data 2. [file elife-89725-fig3-figsupp1-data2.zip › YAP signal.tif]

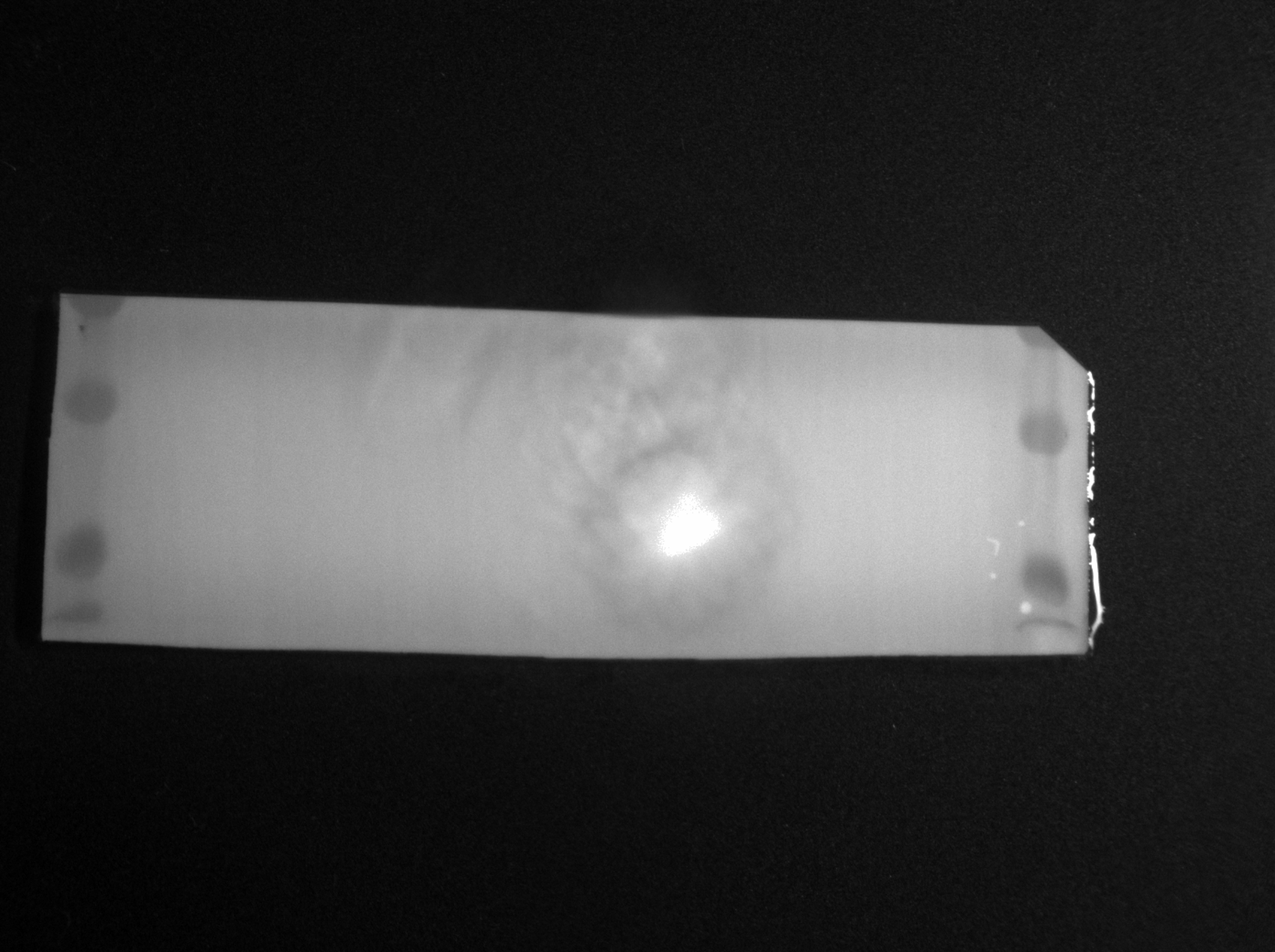

Supplement: Figure 3—figure supplement 1—source data 2. [file elife-89725-fig3-figsupp1-data2.zip › GAPDH ladder.tif]

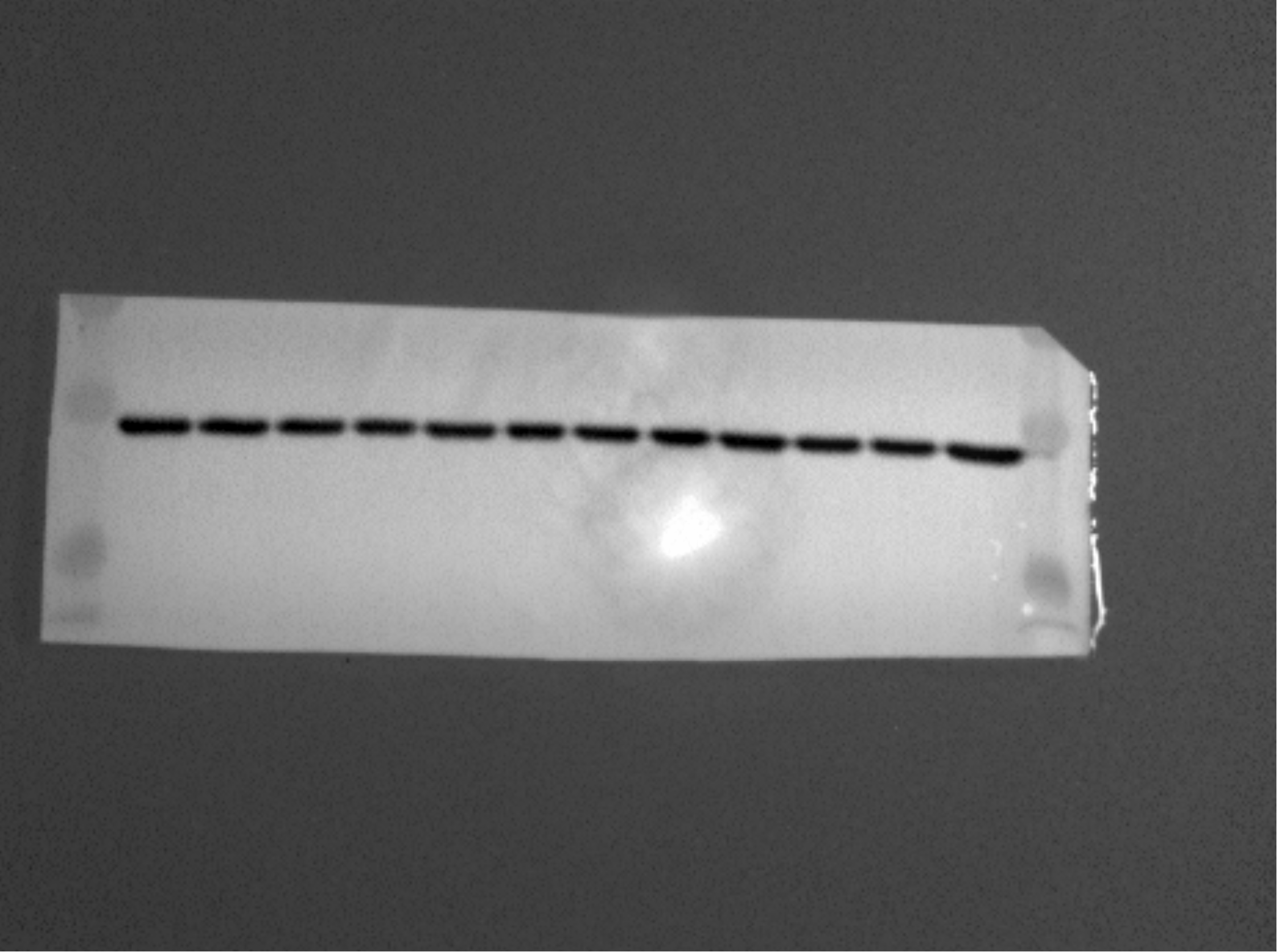

Supplement: Figure 3—figure supplement 1—source data 2. [file elife-89725-fig3-figsupp1-data2.zip › GAPDH merge.tif]

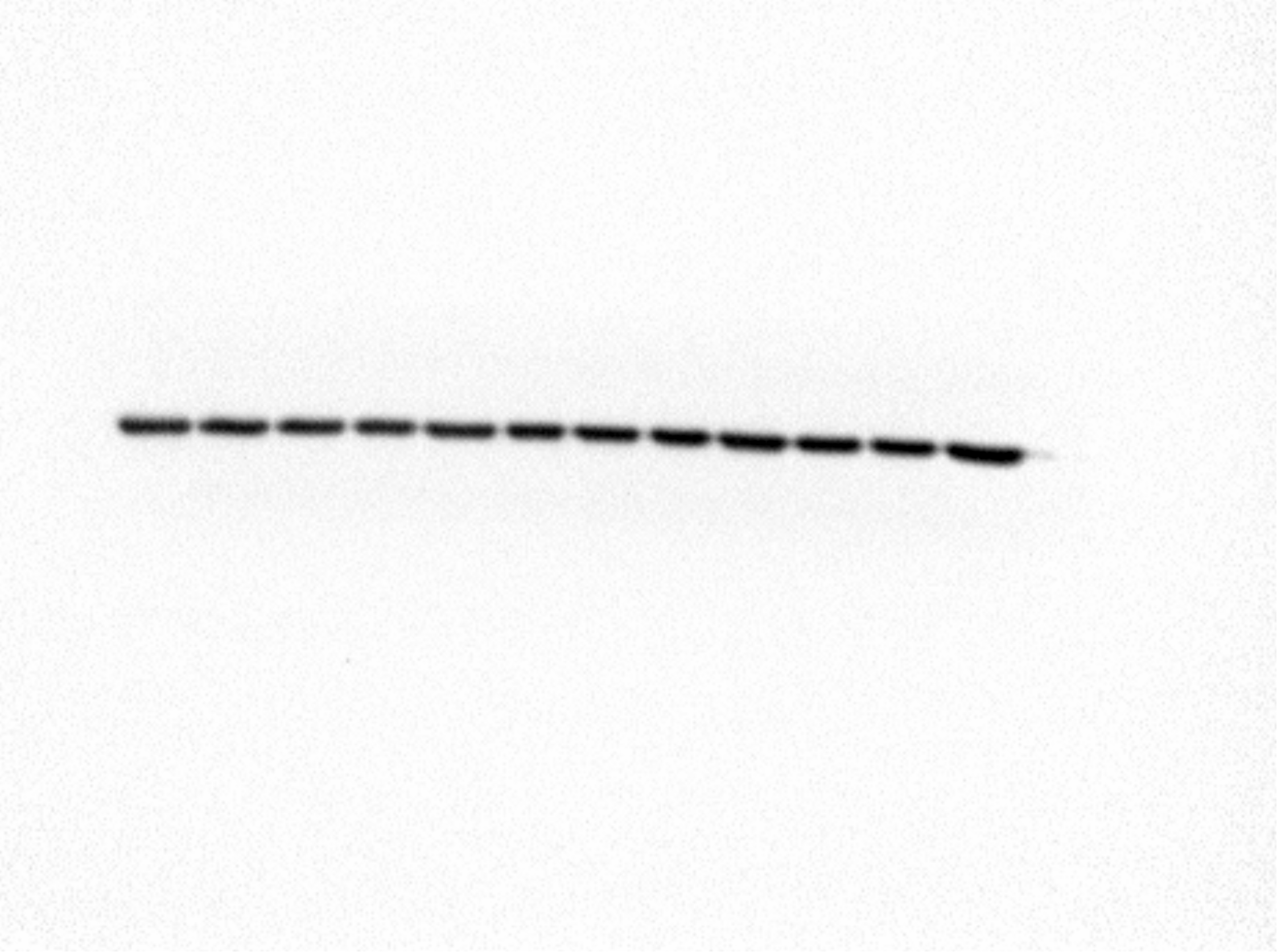

Supplement: Figure 3—figure supplement 1—source data 2. [file elife-89725-fig3-figsupp1-data2.zip › GAPDH signal.tif]
